# Reticuloendotheliosis Virus Inhibits the Immune Response Acting on Lymphocytes from Peripheral Blood of Chicken

**DOI:** 10.3389/fphys.2018.00004

**Published:** 2018-01-23

**Authors:** Yulin Bi, Lu Xu, Lingling Qiu, Shasha Wang, Xiangping Liu, Yani Zhang, Yang Chen, Yang Zhang, Qi Xu, Guobin Chang, Guohong Chen

**Affiliations:** ^1^Department of Animal Genetics and Breeding, College of Animal Science and Technology, Yangzhou University, Yangzhou, China; ^2^Department of Poultry Genetics and Breeding, Poultry Institute, Chinese Academy of Agricultural Sciences, Yangzhou, China

**Keywords:** REV, chicken lymphocyte, DGEs and pathway, cell proliferation, immune response

## Abstract

Chicken reticuloendotheliosis virus (REV) causes the atrophy of immune organs and immuno-suppression. The pathogenic mechanisms of REV are poorly understood. The aim of this study was to use RNA sequencing to analyse the effect of REV on immunity and cell proliferation in chicken lymphocytes from peripheral blood *in vitro*. Overall, 2977 differentially expressed genes (DEGs) were examined from cells between infected with REV or no; 56 DEGs related to cell proliferation and 130 DEGs related to immunity were identified. MTT, Q-PCR, and FCM indicated that REV reduced the number of lymphocytes by inhibiting the transition of S/G1 phase through FOXO and p53 pathways. Similarly, REV infection would destroy the immune defense of lymphocytes through MAPK-AP1 via Toll-like receptor-, NOD-like receptor-, and salmonella infection pathways to reduce the secretion of IL8 and IL18. In addition, the reduction of lymphocytes also might be responsible for the lower levels of IL8 and IL18, and the rescue of lymphocytes would been activated still through FOXO and p53 pathways. Moreover, the immune response for REV in lymphocytes would activate by up-regulating the expression of *NOD1, MYD88*, and *AP1* through Toll-like receptor-/NOD-like receptor/salmonella-MAPK-AP1 pathways. These results indicate that REV could affect lymphocytes from peripheral blood by inhibit the cell proliferation and the immune system. It also was revealed that *NOD1, MYD88*, and *AP1* were the key genes to activate the immune response through Toll-like receptor-/NOD-like receptor/salmonella-MAPK-AP1 pathways. These findings establish the groundwork and provide new clues for deciphering the molecular mechanism underlying REV infection in chickens.

## Introduction

Chicken reticuloendotheliosis is a serious infectious oncosis caused by reticuloendotheliosis virus (REV) infected lymphocytes or reticuloendothelial cells (Witter et al., 1979). Many studies have been reported on the epidemiology, vaccines, and the pathogenesis of REV (García et al., 2003; Fadly and Garcia, 2006; Cheng et al., 2007; Li and Cui, 2007). REV causes the atrophy of immune organs (thymus and bursa of Fabricius) leading to immuno-suppression (Witter et al., 1981; Nazerian et al., 1982). Previous studies have demonstrated a change in IL-2 secretion. T lymphocytes in the thymus and spleen were damaged by REV causing a reduction in the secretion levels of IL-2 (Hrdlicková et al., 1994). A decrease in IL-2 secretion further reduced the number of T cells, and caused serious immune dysfunction when secreting positive immune cytokines to influence the differentiation of T cells into TH and CTL cells, which have different functions (Li and Liu, 1999; Kim et al., 2004).

Although a certain studies on the mechanism of REV pathogenesis and immunity have focused on genome and proteome levels (Li et al., 2016; Xue et al., 2017), these processes are still poorly understood on gene expression level. This study had systematically identified the global genes and pathways by RNA sequencing in chicken lymphocytes after REV infection. The present study investigated the effect of REV on chicken blood lymphocytes.

## Materials and methods

### Animals and ethics statement

Experimental chickens at 21 day were from a pure line of SPF Rugao chicken from the Poultry Institute, Chinese Academy of Agricultural Sciences. Blood was collected from the wing vein, using citric acid dextrose as an anticoagulant for subsequent lymphocytes derived. This study was carried out in strict accordance with the recommendations in the Guide for the Care and Use of Laboratory Animals of the Ministry of Science and Technology of the People's Republic of China (2006).

All experimental procedures were performed in accordance with the Administration Act of Experimental Animals using and care in Jiangsu Province (#115th Jiangsu Province Government Notice in 2008). All of the animal experimental operations were approved and guided by the Animal Care and Use Committee of Yangzhou University.

### The standard biosecurity or institutional safety procedures

All experiments with the REV viruses *in vivo* and *vitro* were conducted in a key biosafety Laboratory of Animal Genetics and Breeding of Molecular Design of Jiangsu Province.

The half lethal dose and the minimum lethal dose of biological agents involved in the experiment were tested by the test center of Yangzhou University (Certificate Number: 150000002432), the doses of biological agents used in experiment are within half the amount of lethal dose, normal animals do not die, which were approved by the laboratory management of Yangzhou University.

### Lymphocytes culture and REV infection

Lymphocytes were derived from chicken blood according the protocol of the chicken blood lymphocyte separation medium kit. Cells (5 × 10^5^/ ml) were incubated with RPMI-1640 medium containing 10% FBS in 10- cm dishes for 24 h, and then infected with reticuloendotheliosis virus strain HA1101 (REV, GenBank accession number: KF305089.1) with 10^5^ TCID_50_/0.1 ml coming from the Key Laboratory of Jiangsu Preventive Veterinary Medicine. Cells were harvested for use in RNA sequencing, FCM, Q-PCR, and ELISA after infection for 36 h. Another set of eight independent replicates were subjected to the same treatment for MTT analysis. Other studies in lymphocytes were carried out in triplicate.

### RNA sequencing analysis

Total RNA was isolated from REV infected or uninfected cells using Trizol reagent (Invitrogen, Carlsbad, California, USA) according to the manufacturer's instructions and dissolved in RNase-free water at a final concentration, 2.0 μg/μl. RNA sequencing was performed by Anno-road Genomics Company (Beijing, China), and data extraction was carried out following the standard protocol (Liao et al., 2013; Love et al., 2014). Genes were considered differentially expressed genes (DEGs) only when the fold-change in abundance for all comparisons exceeded 2.0, with a *P*-value < 0.05. Using the Euclidian metric, average linkage hierarchical clustering was performed based on DEGs. In heat-maps, the color of features was determined by log2 (reference/sample).

### Gene ontology (GO) and kyoto encyclopedia of genes and genomes (KEGG) analysis

Based on the DEGs, Gene Ontology enrichment analysis was performed using the GOEAST software toolkit. The significance level of GO term enrichment was set as FDR-adjusted *p*-value < 0.05 by the Yekutieli method. Enriched KEGG pathways with DEGs were identified by a hypergeometric test using R packages (*P* < 0.01, FDR adjusted). Pathways with < 3 known chicken genes were discarded. Graphical pathway maps were downloaded from the KEGG FTP server, and DEGs were then highlighted in them according to the coordinate description in the XML files at the KEGG FTP server, using Perl GD, XML::Parser and XML::LibXML modules.

### Validation by quantitative polymerase chain reaction (Q-PCR)

Primer information is listed in Table S1. Each 20 μl PCR mixture contained 10 μl of the 2 × iQ™ SYBR Green Supermix, 0.5 μl (10 mM) of each primer and 1 μl of cDNA. Mixtures were incubated in a Real-Time PCR Detection System (ABI7500, Carlsbad, California, USA). A melting curve was constructed to verify that only a single PCR product was amplified. Samples were assayed in triplicate with standard deviations of threshold cycle (CT) values not exceeding 0.5 on a within-run basis. Correlation analysis for gene expression between the two methods was performed.

### Screen of related DEGs

Based on DEGs, gene ontology (GO) enrichment analysis was performed using the GOEAST software toolkit, and the significance level of GO term enrichment was set as a FDR-adjusted *p*-value < 0.05 by the Yekutieli method. DEG-related focus traits were screened.

### Cell proliferation assay

Lymphocytes in six-well culture dishes were infected with REV or were uninfected, in RPMI1640 medium containing 10% FBS for 48 h, and then collected by centrifugation at 500 × g for 5 min for MTT detection. For the MTT assay, 20 μl of MTT (5 mg/ml) was added to each well and incubated for 4 h at 37°C. After removing the supernatant, formazan crystals were dissolved in 200 μl DMSO and the absorbance was measured at 490 nm. Each group had eight repeat wells to ensure the accuracy of the experiment.

### Flow cytometry (FCM) assay

The same samples in the cell proliferation assay were used for the cell cycle assay by FCM. Cells were digested by trypsin, collected after 500 × g centrifugation for 5 min and washed with ice-cold PBS. The cell pellet was suspended with 70% ethanol at 4°C overnight, washed with PBS, then incubated with 180 μ g/ml RNase A at 37°C for 15 min. For FCM, 50 μg/ml propidium iodide (final concentration) was added for 15 min staining in the dark at 37°C. Data were plotted and analyzed using FCS Software (De Novo Software, Los Angeles, CA).

### ELISA for immune factors

The cell medium from REV infected or uninfected groups were used for the detection of TNF2, IL8, and IL18, and the assay was performed using a specific ELISA kit (B&D, Minnesota, USA) according to the manufacturer's instructions.

### Statistical analyses

Statistical differences between groups were evaluated using the Student's *t*-test. *P* < 0.05 (^*^) or < 0.01 (^**^) was considered significant. Data are represented as the mean ± SD.

## Results

### Differentially expressed gene profiles in lymphocytes after infection with REV

**Figure 1:**
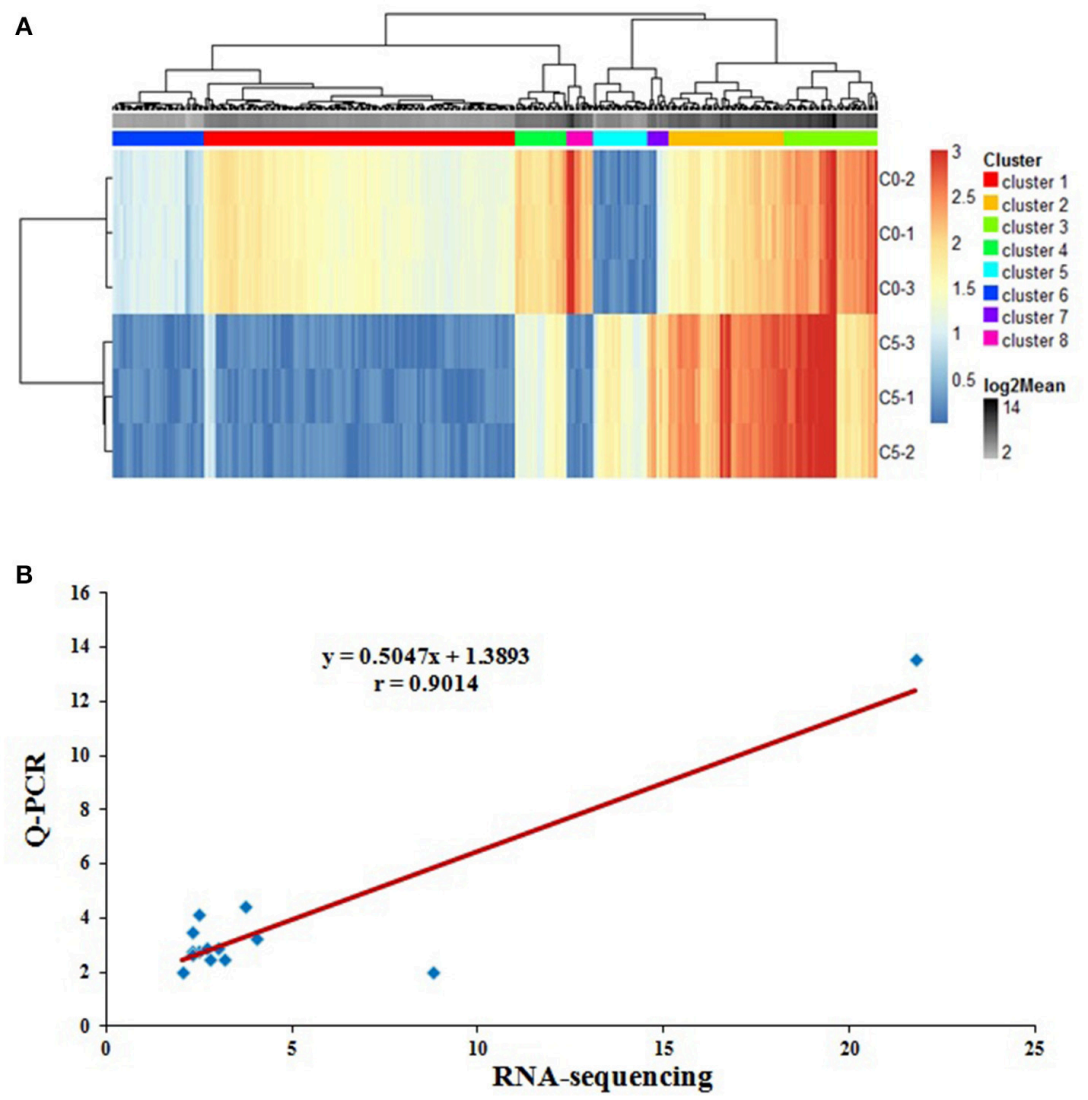
The quality analysis of RNA-sequencing. **(A)** Based on the 2977 known DEGs from RNA sequencing between REV infected or uninfected cells for 36 h, the heat-map of all six samples using Cluster 3.0 software demonstrated that three samples in same group were respectively polymerized; **(B)** Correlation analysis of the fold-changes of 26 DEGs by RNA-sequencing and Q-PCR to validate the results from RNA sequencing. The data was highly correlated (*r* = 0.9014, *P* < 0.01), and the results from RNA sequencing were accurate, *n* = 26.

Using the lymphocytes from peripheral blood infected with REV or not, gene expression profiles were examined using RNA sequencing. 2977 known genes, 1981 down-regulated, and 996 up-regulated (Infection vs. Control), were detected as differentially expressed genes (DEGs) with consistent fold changes ≥2.0 (Table S2). Based on the 2977 known DEGs, a cluster analysis of all six samples was performed by Cluster 3.0 software, three samples in same one group were respectively polymerized (Figure 1A). in addition, some DEGs were selected to examine the relative expression using Q-PCR. As shown in Figure 1B, the fold-changes of 26 genes by RNA-sequencing and Q-PCR were highly correlated (*r* = 0.9014, *P* < 0.01).

GO analysis based on the 2977 DEGs was performed, the enriched GO-terms (*P* < 0.05) were selected (Table S3), including defense response to bacterium, inflammatory response, cell cycle, cell proliferation, etc. By GO-term analysis, 56 known DEGs related to cell cycle (Table S4), and 130 known DEGs related to immunity (Table S5) were screened. After KEGG pathway analysis, 14 metabolic pathways were screened (Table S6). As expected, some well-known pathways were enriched, which affect immune responses (Toll-like receptor-, NOD-like receptor-, and salmonella infection signaling pathways), cell proliferation or apoptosis pathways (cell cycle- and FOXO signaling), glyco- and lipid- metabolism pathways (Insulin signaling).

### REV infection inhibited the cell proliferation of lymphocyte

**Figure 2:**
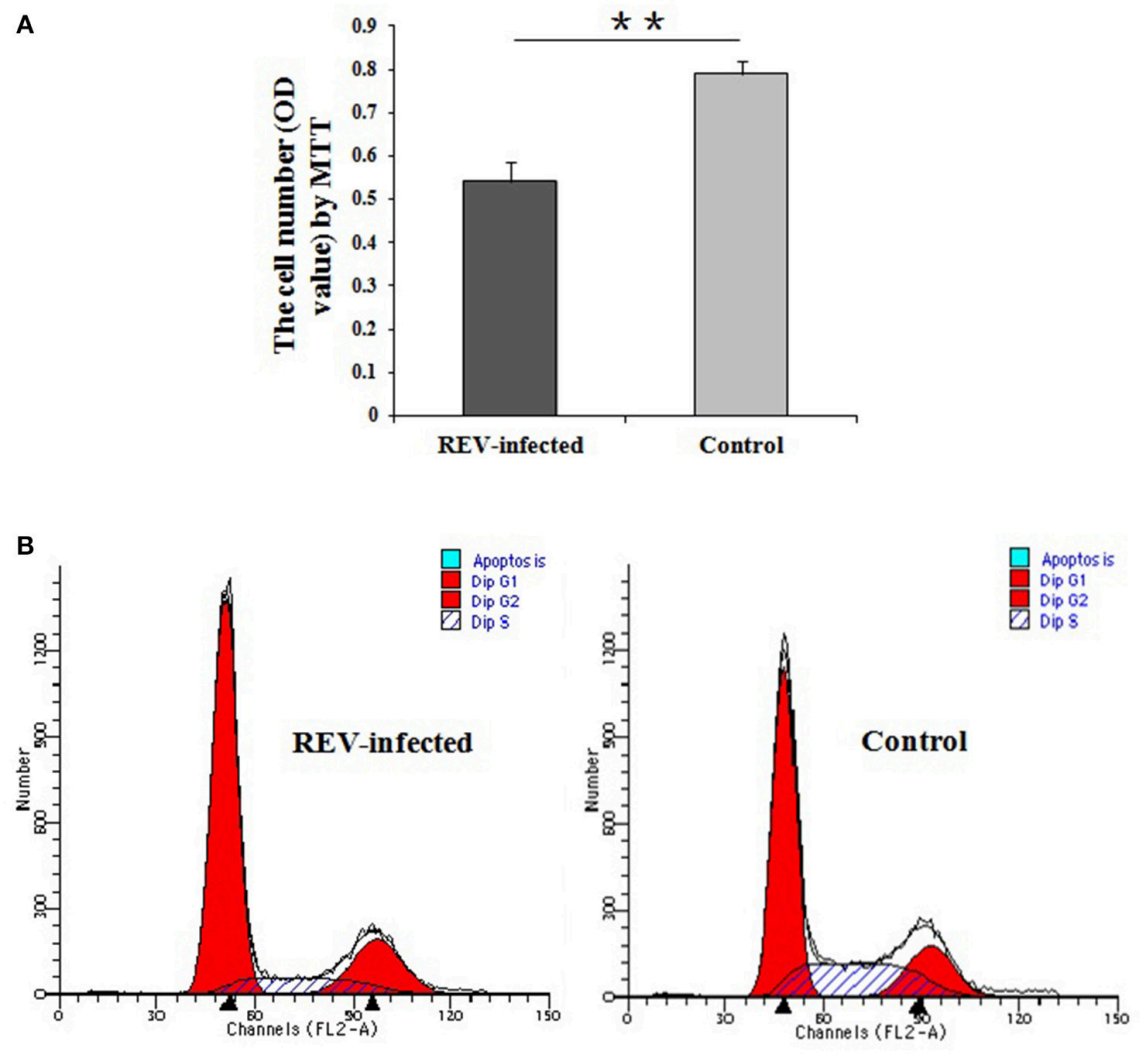
Detection of cell proliferation and cell cycle of lymphocytes infected with REV for 36 h. **(A)** Comparative analysis of cell number in REV infected or uninfected groups by MTT. The cell numbers were significantly reduced in REV infected cells compared with controls (*P* < 0.01). Data are means ± SEM (*n* = 8); **(B)** Cell cycle analysis by FCM. The cell percentage in S phase was significantly lower (*P* < 0.01) and in G1 phase was higher (*P* < 0.01) in REV infected cells compared to the controls. The G1/S transition in lymphocytes was inhibited by REV infection. ^**^ *P* < 0.01.

Cell proliferation detection by MTT showed that lymphocyte numbers were significantly reduced in REV infected cells compared with controls (*P* < 0.01) (Figure 2A). Compared with the controls, the results by FCM showed that the percentage of REV infected cells was significantly lower (*P* < 0.01) in S phase and higher (*P* < 0.01) in the G1 phase (Figure 2B).

**Figure 3:**
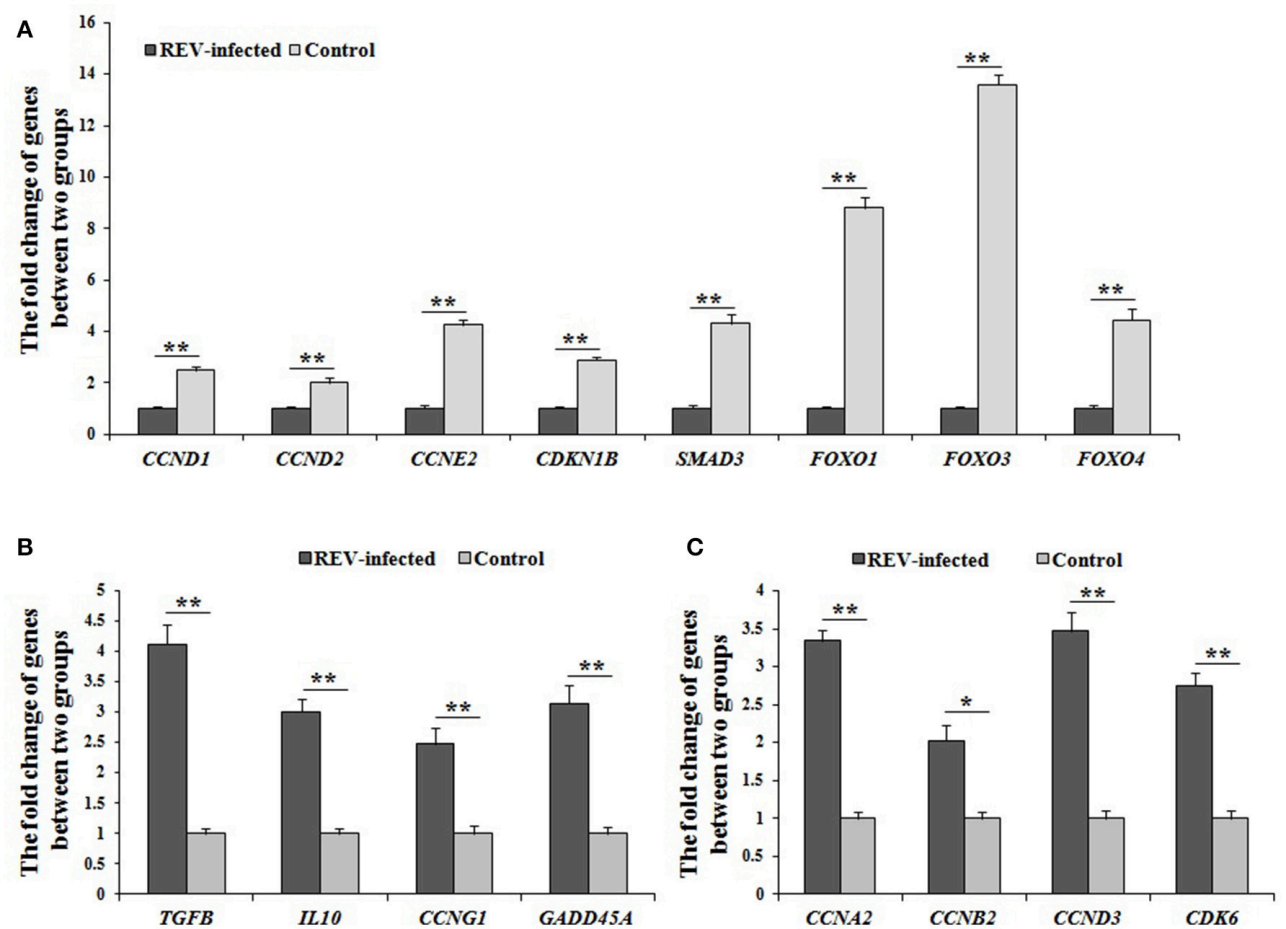
The differential expression of genes related to cell cycle by Q-PCR in REV infected cells. **(A)** The expression levels of *CCND1, CCND2, CCNE2, CDKN1B, SMAD3, FOXO1, FOXO3*, and *FOXO4* were significantly down-regulated (*P* < 0.01) in infected cells compared with controls; **(B)** The expression levels of *TGFB, IL10, CCNG1*, and *GADD45A* were significantly up-regulated (*P* < 0.05 or *P* < 0.01) in infected cells compared with controls; **(C)** The expression levels of *CCNA2, CCNB2, CCND3*, and *CDK6* were significantly up-regulated (*P* < 0.05 or *P* < 0.01) in infected cells compared with controls. Data are means ± SEM (*n* = 3). ^*^P < 0.05, ^**^P < 0.01.

In FOXO signaling pathway (including TGFβ, Insulin, IL10, and MAPK pathways) (Figure S1A), *SMAD3* (the key factor in TGFβ pathway) and *FOXO* (including *FOXO1, FOXO3*, and *FOXO4*, the common key factor in Insulin-, IL10-, and MAPK pathways) were enriched, and would regulate the expression of *CCNB2, CCNB3, CCND1, CCND2, CCND3, CCNG2*, and *CDKN1B*, which involves in the cell cycle. In p53 signaling pathway (Figure S1B), *CCNA2, CCND1, CCND2, CCND3, CCNE2, CCNH, CDKN1B, CDK6, CDK7*, and *GADD45A* were enriched to regulate cell cycle. In addition, *SIAH1* and *WIPI2* were enriched in p53 negative feedback of p53 pathway. By Q-PCR, *SMAD3, FOXO1, FOXO3, FOXO4, CCND1, CCND2, CCNE2*, and *CDKN1B* were significantly down-regulated (*P* < 0.01) (Figure 3A). These results strongly supported that REV would inhibit the cell proliferation of lymphocyte from chicken peripheral blood through p53 and FOXO pathways.

However, the confusing results were also found in this study. As the starting factors of TGFβ-, IL10-, MAPK-, and p53- pathways, the expressions of *TGFB, TGFBR1, TGFBR2, IL10, Grb2, CCNG1*, and *GADD45A* were significantly up-regulated in REV infected cells compared with controls, and the expression of *TGFB, IL10, CCNG1*, and *GADD45A* by Q-PCR was same with the data by RNA-sequencing (Figure 3B). In addition, the expression levels of *CCNA2, CCNB2, CCND3*, and *CDK6* were also significantly up-regulated (*P* < 0.05 or *P* < 0.01) in REV infected cells compared with controls (Figure 3C).

### The immune damage and response synchronously occurs by MAPK-AP1 pathway in lymphocytes for REV

As lymphocytes were used, it is reasonable to assume that DEGs in the current study contributed to immune responses after REV infection, and the representative immune factors also were screened. Some genes involved in *ILs* and their receptors were differentially expressed by RNA sequencing. The expression levels of *IL8L1, IL15, IL16, IL18, IL1R, IL2R*, and *IL18R* were significantly down-regulated in infected cells. Similarly, several genes of the TNF super-family (*TNFSF8, TNFSF10, TNFSF13B*, and *TNFSF15*) also were found from RNA sequencing data. Except for *TNFSF10*, their expression levels were down-regulated by REV. Moreover, *TLR2A, TLR4, TLR7, TLR15* genes also were identified as DEGs, and their mRNA levels were significantly down-regulated in infected cells.

**Figure 4:**
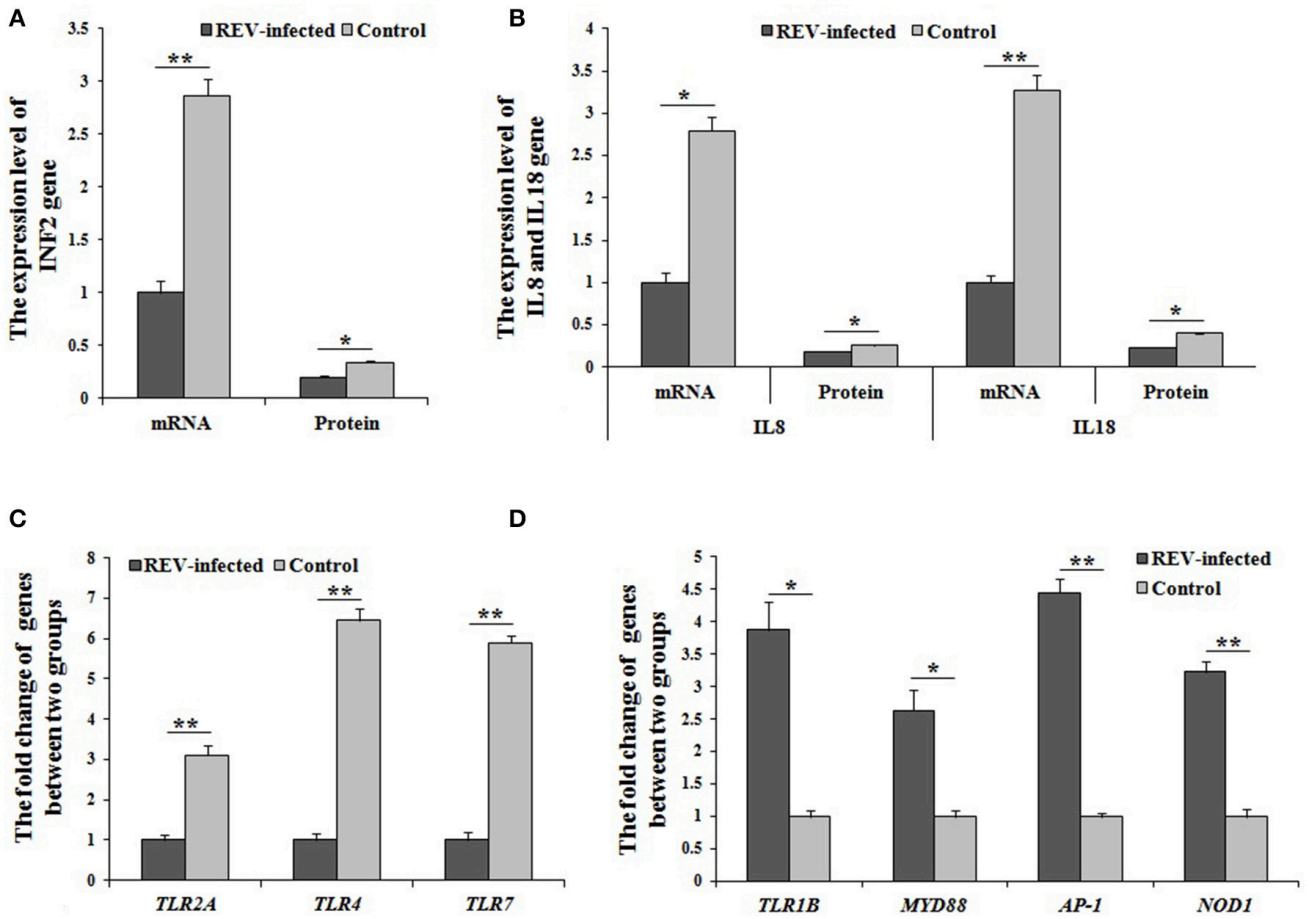
The mRNA or protein levels of genes related to immunity by Q-PCR or ELISA. **(A,B)** The mRNA and protein levels of TNF2, IL8, and IL18 were significantly lower (*P* < 0.05 or *P* < 0.01) in infected cells compared with controls; The expression levels of cluster of differentiation (CD) genes were significantly up-regulated (*P* < 0.05 or *P* < 0.01) in infected cells compared with controls; **(C)** The mRNA levels of *TLR2A, TLR4*, and *TLR7* were significantly changed (*P* < 0.01) between the two groups; **(D)** The mRNA levels of *TLR1B, MYD88, AP-1*, and *NOD1* were significantly changed (*P* < 0.05 or *P* < 0.01) between the two groups. Data are means ± SEM (*n* = 3).

Combined with the results of the KEGG analysis, the identified DEGs related to immune defense were enriched in salmonella infection-, NOD-like receptor-, and Toll-like receptor pathways. In the Toll-like receptor pathway (Figure S2A), a decrease in *TLR2A, TLR4*, and *TLR7* through MAPK-AP-1 pathway, down-regulated the transcript abundance of *IL8* to reduce chemotaxis, T cell stimulation and antiviral effects. For NOD-like receptor pathway (Figure S2B), the transcript abundance of *IL8, IL18*, and *IFN* were regulated MAPK-AP-1 pathway and reduced the immune actions of pro-inflammatory cytokines and chemokines. In salmonella infection pathway (Figure S2C), *IL8* and *IL18* genes down-regulated the expression of through MAPK-AP-1 pathway. Among the factors, TNF2 gene was identified, and had significantly lower mRNA and protein levels (*P* < 0.01, *P* < 0.05, respectively) (Figure 4A). Similarly, IL8 and IL18 genes also had significantly lower mRNA and protein levels (*P* < 0.01) in infected cells compared with controls (Figure 4B). Moreover, *TLR2A, TLR4*, and *TLR7* genes also had significantly lower mRNA and protein levels (*P* < 0.01) in infected cells compared with controls (Figure 4C). Overall the above results, REV would reduce the secretion of IL8 and/or IL18 through three (salmonella infection-, NOD-like receptor-, and Toll-like receptor pathways), which would together in MAPK-AP1 pathway.

Based on the identified salmonella infection-, NOD-like receptor-, and Toll-like receptor pathways related to immunity, some important in immune genes were screened as DEGs by RNA-sequencing. In Toll-like receptor pathway, *TLR1B, MYD88*, and *AP-1* were significantly up-regulated (*P* < 0.05 or *P* < 0.01) in REV infected cells compared with control cells by Q-PCR (Figure 4D). For the NOD-like receptor pathway, *NOD1* and *AP-1* were significantly up-regulated (*P* < 0.01) in REV infected cells compared with control cells by Q-PCR (Figure 4D). In the salmonella infection signaling pathway, *MYD88* and *AP-1* were significantly up-regulated (*P* < 0.05 or *P* < 0.01) in REV infected cells compared with control cells by Q-PCR (Figure 4D).

## Discussion

Chicken REV infected lymphocytes or reticuloendothelial cells, and caused the atrophy of immune organs (thymus and bursa of Fabricius) leading to immuno-suppression (Witter et al., 1981; Nazerian et al., 1982). Many studies of REV regarding epidemiology, vaccines, and the mechanism of pathogenesis and immunity have been reported (García et al., 2003; Fadly and Garcia, 2006; Cheng et al., 2007; Li and Cui, 2007), however is still lacking the systematically undertaken research on molecular regulation mechanism and immune response for REV in chicken lymphocytes from peripheral blood. The objective of this study was to investigate the effect of REV on chicken blood lymphocytes by RNA sequencing. Potential candidate related DEGs were defined from the data of RNA sequencing using different RNA samples (*n* = 3 dishes), with a required fold change in expression of ≥2.0. The cluster analysis of six RNA samples was performed, three samples in the same group were respectively polymerized, so the comparability among groups was believed. To deeply confirm the obtained data by RNA sequencing, 156 tests of 26 related DEGs were performed between two groups by Q-PCR, and the relationship on fold-changes of gene expression by two methods were significantly correlated (*r* = 0.9014), indicating that the accuracy of data from RNA sequencing was verified in this study.

It was reported that REV infection induced a splenic suppressor cell population which cytostatically inhibited the proliferation of cytotoxic cells capable of lysing REV-T tumor cells (Bose, 1984). A reduction of lymphocytes with REV infected *in vitro* was found in this study. Moreover, the results of FCM revealed that the G1/S phase transition was interdicted to inhibit cell proliferation of lymphocytes. Genetically, the FOXO and p53 pathways, which regulate cell proliferation (Zaballos and Santisteban, 2013; Liu et al., 2016; Yi et al., 2016; Tang et al., 2017), were enriched by KEGG analysis. Further, *SMAD3* and *FOXO* (including *FOXO1, FOXO3*, and *FOXO4*) were involved in FOXO pathway (including TGFβ, Insulin, IL10, and MAPK pathways), and would regulate the expression of *CCNB2, CCNB3, CCND1, CCND2, CCND3, CCNG2*, and *CDKN1B* in this study. Similarly, *CCNA2, CCND1, CCND2, CCND3, CCNE2, CCNH, CDKN1B, CDK6, CDK7*, and *GADD45A* were involved in p53 pathway. In addition, *SIAH1* and *WIPI2* were enriched in p53 negative feedback. In overall consideration of above results and known information (Lee et al., 2012; Hill et al., 2014; Zhao et al., 2015; Vezzali et al., 2016), genes (*SMAD3, FOXO1, FOXO3, FOXO4, CCND1, CCND2, CCNE2, CDKN1B*) were validated by Q-PCR, and their expressions were significantly down-regulated (*P* < 0.05 or *P* < 0.01). So it was strongly supported that REV would inhibit the cell proliferation of lymphocyte, and the key genes (*SMAD3, FOXO1, FOXO3, FOXO4, CCND1, CCND2, CCNE2, CDKN1B)* and the pathways (p53 and FOXO) were considered with the important role in inhibiting cell proliferation of lymphocyte from chicken peripheral blood for REV infection in this study. However, the expression levels of the starting factors (*TGFB, TGFBR1, TGFBR2, IL10, Grb2, CCNG1*, and *GADD45A*) in FOXO and p53 pathways, and genes related to cell cycle (*CCNA2, CCNB2, CCND3, CDK6*) were up-regulated (*P* < 0.05 or *P* < 0.01) in REV infected cells compared with controls by RNA-sequencing and Q-PCR. So it was speculated that the rescue of lymphocyte would activate through FOXO and p53 pathways.

This reduction of lymphocytes caused serious immune dysfunction in secreting immune cytokines that influence the differentiation of T cells into TH and CTL cells (Li and Liu, 1999; Kim et al., 2004). After infected by REV, DEGs contributed to immune responses also were screened, including *ILs, ILRs, TNFs, IFNs*, and *TLRs*, and the expression levels of these genes were significantly down-regulated in infected cells. It was indicated that the immune defense was destroyed for REV infection, and this view was same as previous reported (Witter et al., 1981; Nazerian et al., 1982). Combined with the results of the KEGG analysis, the identified DEGs related to immune responses were enriched in Toll-like receptor-, NOD-like receptor- and salmonella infection signaling pathways, which involved in the process of immune responses (Wong et al., 2009; Coutermarsh-Ott et al., 2016; Huang et al., 2016; Yin et al., 2017). For three enriched pathways (salmonella infection, NOD-like receptor and Toll-like receptor) would directly connect MAPK pathway and AP-1 in this study. Many studies have reported on the MAPK signaling pathway and AP-1 in the regulation of immune factor secretion (Shi et al., 2014; Jiang et al., 2015; Lanna et al., 2017). Moreover, *TLR2, TLR4, TLR7*, and *IL8* were enriched in Toll-like receptor pathway, *IL8* and *IL18* were enriched in the NOD-like receptor pathway, and *TLR4, IL8*, and *IL18* were enriched in the salmonella infection pathway. By Q-PCR, *TLR2, TLR4, TLR7, IL8*, and *IL18* were significantly down-regulated in REV infected cells compared with controls. Further, the protein levels of TNF2, IL8, and IL18 were also detected, and were significantly lower for REV infection. Considered to the function of TLR2, TLR4, TLR7, TNF2, IL8, and IL18 (Briend et al., 2017; Sanguinete et al., 2017; Zhang et al., 2017), it was revealed that the immune defense would been inhibited for REV infection through salmonella infection, NOD-like receptor, Toll-like receptor and MAPK-AP1 pathways. Together all, REV infection would decrease the secretion of immune factors for the reduction of lymphocytes and inhibition of immune defense.

Based on the identified salmonella infection-, NOD-like receptor-, Toll-like receptor, and MAPK-AP1 pathways, *TLR1B, MYD88, NOD1, AP-1*, which had the important role in regulation the immune action (Buchholz and Stephens, 2008; Ramasamy et al., 2012; Limoge et al., 2017; Wang et al., 2017), were significantly up-regulated in REV infected cells compared with control cells by RNA-sequencing and Q-PCR. In addition, *CD40* and *CD80* also were significantly up-regulated in REV infected cells compared with control cells by two methods.

Among them, *NOD1* and *AP-1* were involved in NOD-like receptor pathway. *TLR1B, MYD88, CD40, CD80*, and *AP-1* were involved in Toll-like receptor pathway. *MYD88* and *AP-1* were involved in salmonella infection pathway. *NOD1, TLR1B, MYD88, AP-1*. These results revealed that the immune response for REV would been activated by up-regulating the expression levels of genes (*TLR1B, MYD88, NOD1*, and *AP-1*) through salmonella infection-, NOD-like receptor-, Toll-like receptor, and MAPK-AP1 pathways.

**Figure 5:**
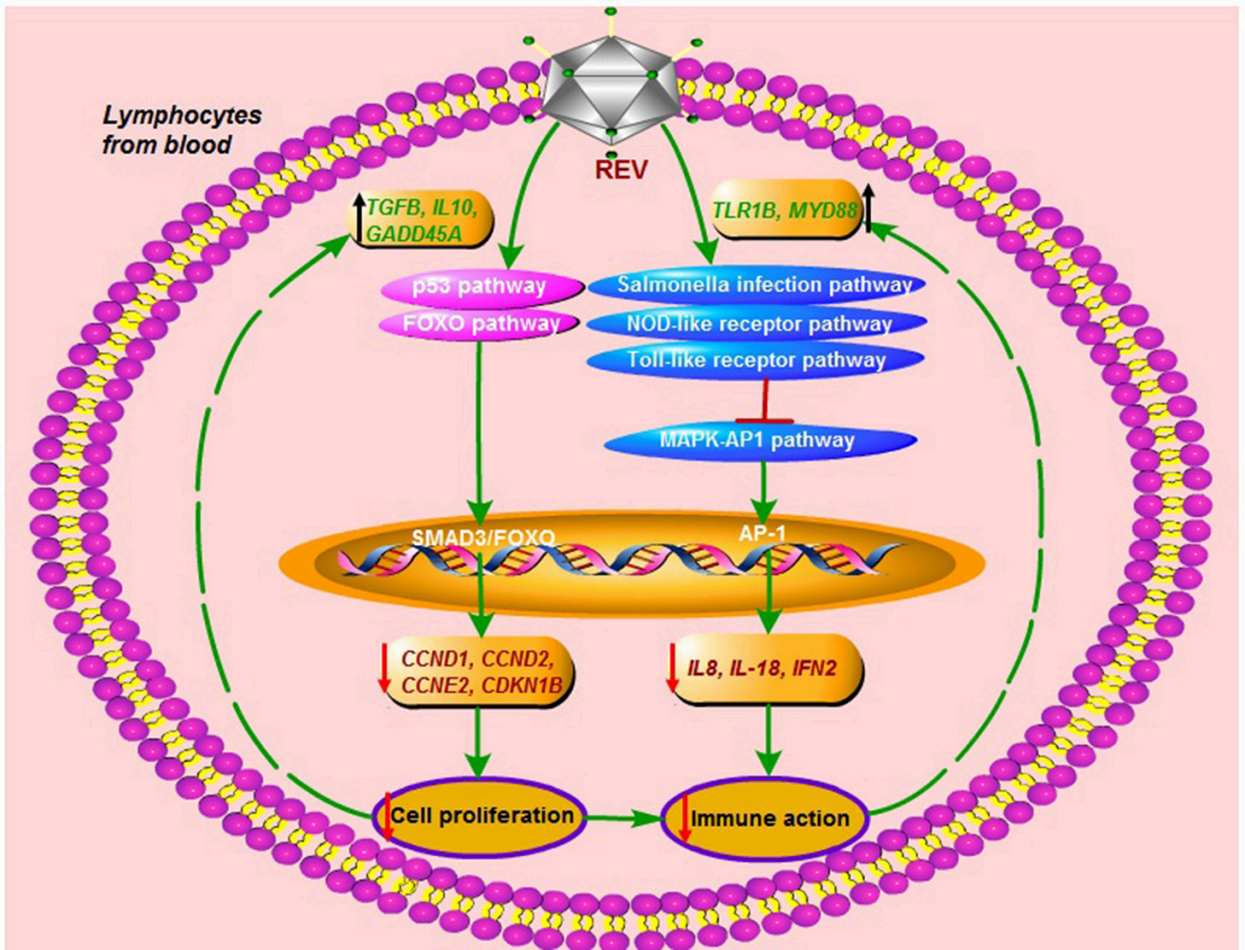
The immune destroy and regulatory mechanism in lymphocytes from chicken peripheral blood for REV infection.

In summary, we report the effect of REV infection on cell proliferation and immune response in chicken peripheral blood lymphocytes *in vitro*. REV infection inhibited the G1/S transition to reduce lymphocyte through p53 and FOXO signaling pathways, and destroyed through salmonella infection-, NOD-like receptor-, Toll-like receptor, and MAPK-AP1 pathways in lymphocytes. On the other hand, the immune response would activate by up-regulating the expression of *TLR1B, MYD88, NOD1*, and *AP-1*. In addition, the immune inhibition of REV was rescued in lymphocytes by activating cell proliferation through p53 and FOXO pathways. Our findings will help to establish the groundwork (Figure 5) and provide new clues for deciphering the molecular mechanisms underlying the pathogenesis of REV in chicken blood lymphocytes. The field of our view was only focus on the transcriptional level of genes, so the additional studies of the post-translational effects were required to complement these mRNA expression analyses.

## Author contributions

GChang, GChen, and YB conceived the project. GChang and YB designed all the experiments. YB, LX, LQ, SW, and XL performed the experiments. YB, YaniZ, YC, YangZ, and QX conducted the bioinformatics analyses. YB contributed to the manuscript preparation.

### Conflict of interest statement

The authors declare that the research was conducted in the absence of any commercial or financial relationships that could be construed as a potential conflict of interest.

## Supplementary material

Table S1 The specific primers for Q-PCR in this study.

Table S2 The differentially expressed gene (DEG) profiles (fold change ≥ 2.0).

Table S3 The enriched GO-terms (based on the 2977 DEGs).

Table S4 The DEGs related to cell cycle and cell proliferation.

Table S5 The DEGs related to immune.

Table S6 The enriched pathways, based on the 2977 DEGs.

Figure S1 The enriched pathways involved in the cell cycle in this study.

Figure S2 The enriched pathways involved in the immunity in this study.

## Abbreviations

CCNs: Cyclins
CD: Cluster of differentiation
CDK: Cyclin-dependent kinases
CT: Threshold cycle
DEGs: Differentially expressed genes
FCM: Flow cytometry
FOXO: Forkhead box O
IFN: Interferon
GO: Gene ontology
IL: Interleukin
KEGG: Kyoto encyc lopedia of genes and genomes
MAPK: Mitogen-activated protein kinase
MYD88: Myeloid differentiation primary response gene 88
MTT: 3-(4, 5-dimethyl-2-thiazolyl)-2,5-diphenyl-2-H-tetrazolium bromide
NOD: Nucleotide-binding oligomer-zation domain
Q-PCR: Quantitative polymerase chain reaction
REV: Reticuloendotheliosis virus
TGF: Transforming growth factor
TLR: Toll-like receptor
TNF: Tumor necrosis factor.
