# Supplementary figures and images for: Reticuloendotheliosis Virus Inhibits the Immune Response Acting on Lymphocytes from Peripheral Blood of Chicken
Source: Front Physiol. 2018 Jan 23;9:4. doi: 10.3389/fphys.2018.00004 (PMC5787092; doi:10.3389/fphys.2018.00004)

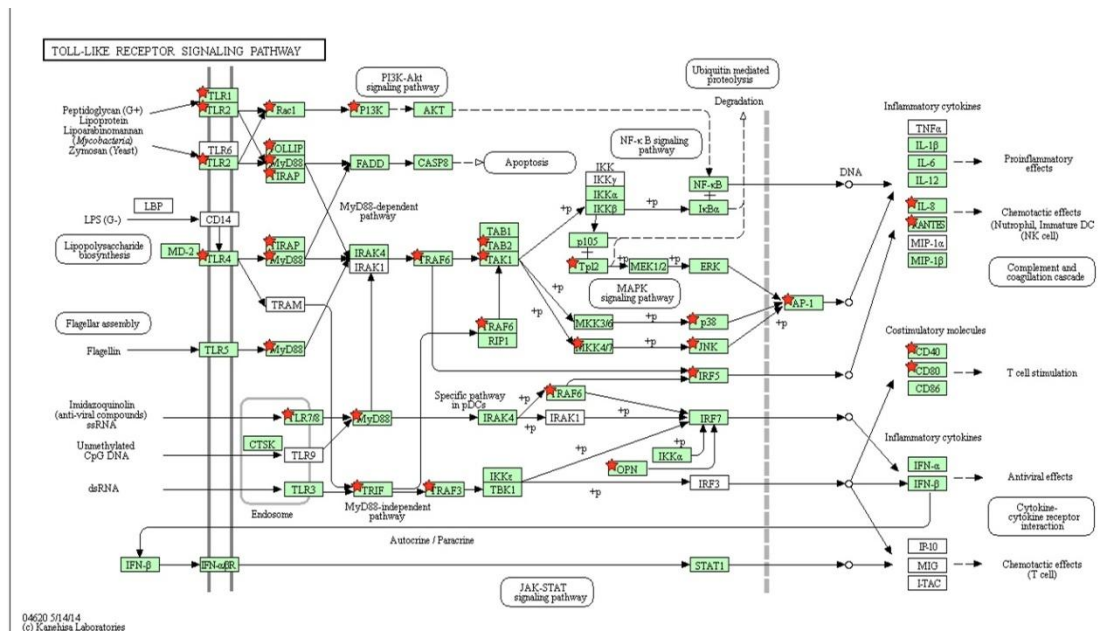

**Fig. S2A**

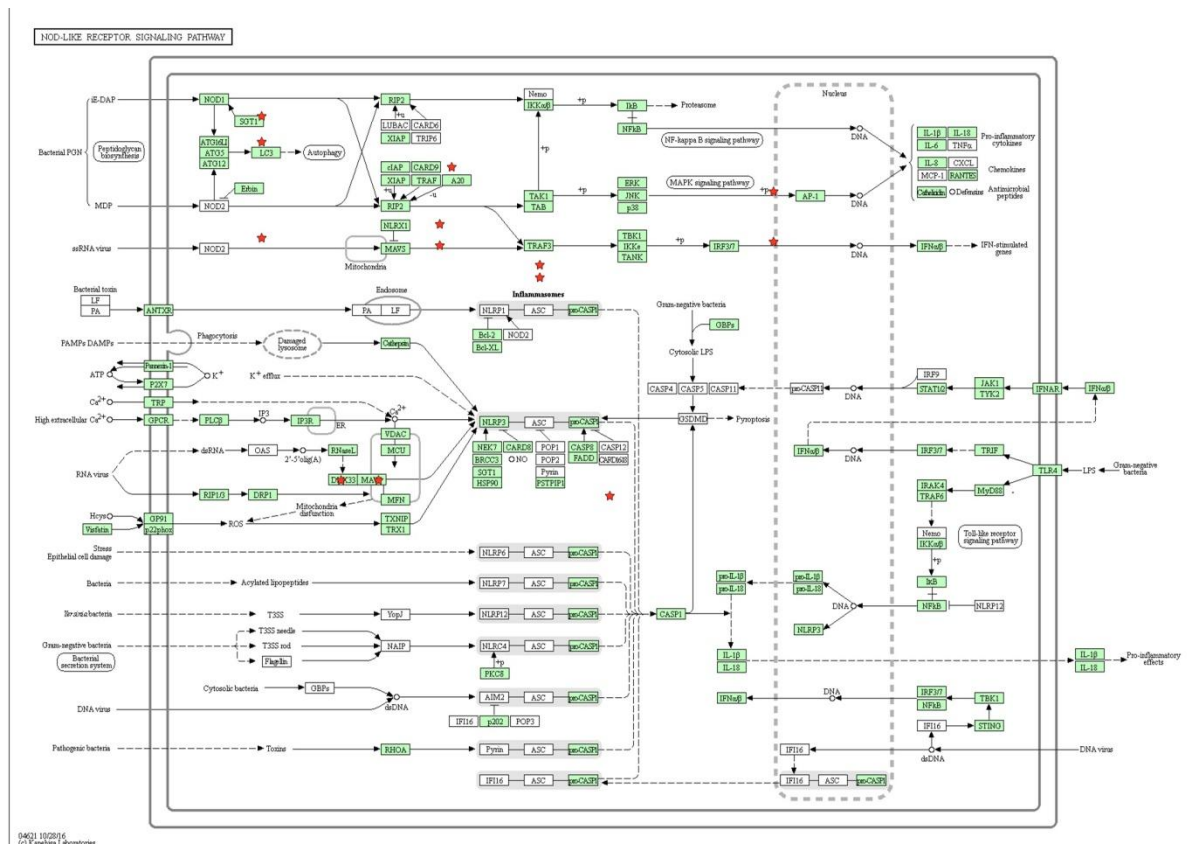

**Fig. S2B**

Supplement: Figure S2 — The enriched pathways involved in the immunity in this study. [file Image2.PDF]
