# Supplementary material for: Reticuloendotheliosis Virus Inhibits the Immune Response Acting on Lymphocytes from Peripheral Blood of Chicken
Source: Front Physiol. 2018 Jan 23;9:4. doi: 10.3389/fphys.2018.00004 (PMC5787092; doi:10.3389/fphys.2018.00004)
Supplement: Figure S1 — The enriched pathways involved in the cell cycle in this study. [file Image1.PDF]

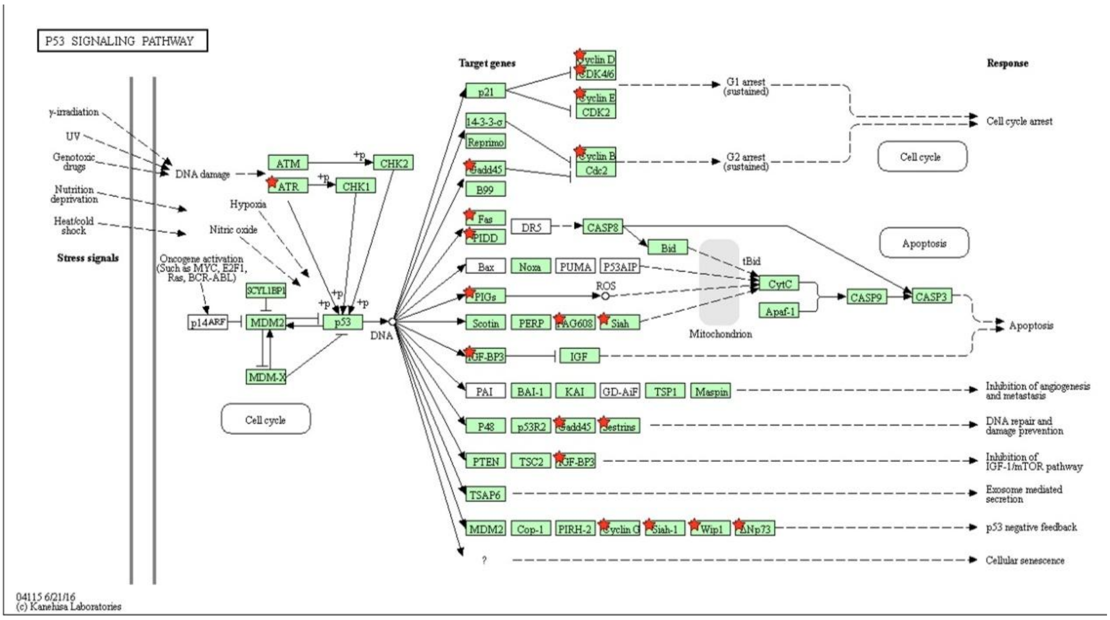

Fig. S1A

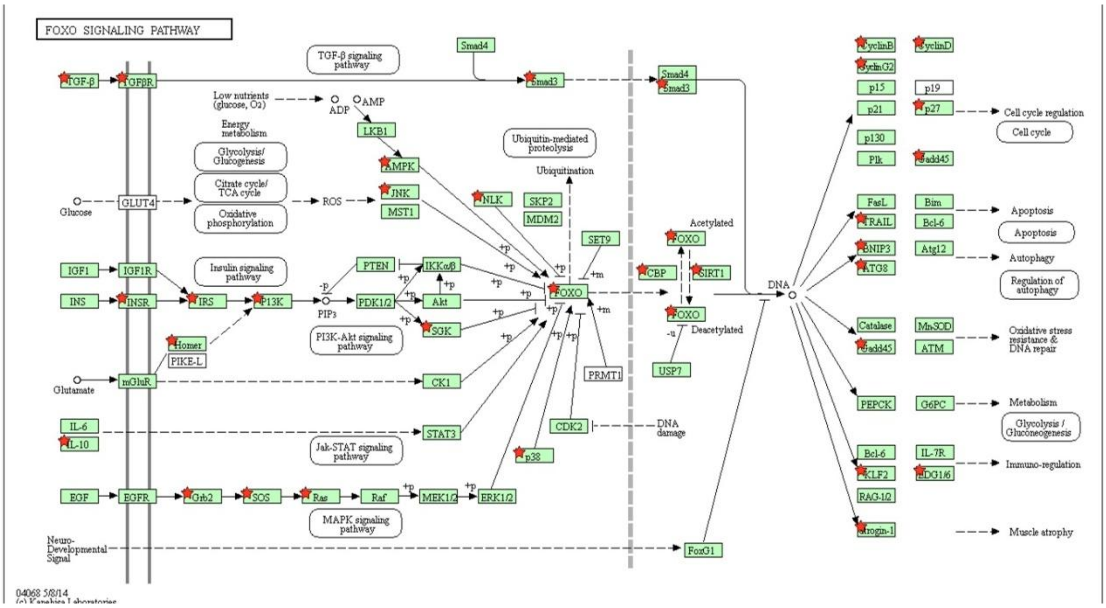

Fig. S1B
